# Itraconazole-Loaded Ufasomes: Evaluation, Characterization, and Anti-Fungal Activity against *Candida albicans*

**DOI:** 10.3390/pharmaceutics15010026

**Published:** 2022-12-21

**Authors:** Sara M. Hashem, Mary K. Gad, Hend M. Anwar, Neveen M. Saleh, Rehab N. Shamma, Noha I. Elsherif

**Affiliations:** 1 National Organization for Drug Control and Research, Cairo 12622, Egypt; 2 Department of Biochemistry, Egyptian Drug Authority, Cairo 12611, Egypt; 3 Department of Microbiology, Egyptian Drug Authority, Cairo 12611, Egypt; 4 Department of Pharmaceutics and Industrial Pharmacy, Faculty of Pharmacy, Cairo University, Cairo 11562, Egypt; 5 Department of Pharmaceutics and Pharmaceutical Technology, Faculty of Pharmacy, Heliopolis University, Cairo 11785, Egypt

**Keywords:** itraconazole, ufasomes, oleic acid, *Candida albicans*, microbiology

## Abstract

Numerous obstacles challenge the treatment of fungal infections, including the uprising resistance and the low penetration of available drugs. One of the main active agents against fungal infections is itraconazole (ITZ), with activity against a broad spectrum of fungi while having few side effects. The aim of this study was to design ufasomes, oleic acid-based colloidal carriers, that could encapsulate ITZ to improve its penetration power. Employing a 2^2^3^1^ factorial design, the effect of three independent factors (oleic acid amount, cholesterol concentration, and ITZ amount) was investigated and evaluated for the percentage encapsulation efficiency (%EE), particle size (PS), and zeta potential (ZP). Optimization was performed using Design^®^ expert software and the optimized ITZ-loaded ufasomes obtained had %EE of 99.4 ± 0.7%, PS of 190 ± 1 nm, and ZP of −81.6 ± 0.4 mV, with spherical unilamellar morphology and no aggregation. An in vitro microbiological study was conducted to identify the minimum inhibitory concentration of the selected formula against *Candida albicans*, which was found to be 0.0625 μg/mL. Moreover, the optimized formula reduced the expression of toll-like receptors-4 and pro-inflammatory cytokine IL-1β secretion in the *C. albicans*-infected fibroblasts, indicating that the proposed ITZ-loaded ufasomes are a promising drug delivery system for ITZ.

## 1. Introduction

Parasitizing a human cell is a complex process for a fungus [1], as it needs four conditions to be satisfied, which are its capability to grow at a temperature above 37 °C, the ability to reach the tissues that will be parasitized, the ability to absorb human tissue components, and finally being resistant to the immunological system of humans [2,3]; and thus the role of virulence factors is highlighted. Virulence factors are necessary for the fungi survival [4], as well as their ability to resist antifungal agents [5]. *Candida albicans* is thought of as a dangerous opportunistic pathogen that causes, in the United States of America, 6.8% of hospital-acquired infections [6]. Regardless of the wide range of antifungal drugs, invasive candidiasis remains a challenge in terms of the mortality rates and hospitalization time [7,8]. Moreover, the urgency of resistance to the approved antifungals increases the gravity of this problem [9]. Consequently, humans need to develop new scaffolds for antifungals that can combat this resistance.

One of the antifungal agents effective against *C. albicans* is itraconazole (ITZ) [10]. ITZ exerts its action via different mechanisms. It inhibits the lanosterol 14α-demethylase, which leads to the inhibition of the synthesis of fungal-mediated ergosterol [11]. Uniquely, ITZ inhibits the hedgehog signaling pathway and angiogenesis, linking ITZ to the inhibition of glycosylation, VEGFR2 phosphorylation, trafficking, and cholesterol biosynthesis [12,13]. Due to its effectiveness, ITZ is formulated in several systemic oral marketed products such as Sporanox^®^ and Fungitraxx^®^ [14]; however, no topical marketed products are available. Several studies have tried to encapsulate ITZ in several novel drug delivery systems, to benefit from its advantages, including chitosan nanoparticles [15], microneedle patches [16], nanoemulsion formulations [17], nanoporous silica xerogel [18], and nanostructured lipid carriers [19]. Chemically, ITZ is a lipophilic drug with a partition coefficient (n-octanol/aqueous buffer) k = 5.66 [20]; however, ITZ has a low permeability [19]. The aim of this study was to enhance the permeability of ITZ topically by encapsulating it in a colloidal carrier.

Ufasomes are colloidal carriers, formed of fatty acids with their ionized species in an alkali medium (pH 7–9) to form a suspension of closed lipid bilayers [21]. Ufasomes were first used in 1973 by Gebicki and Hicks [22], who described the ufasomes and their stability. The stability of ufasomes depends on the selection of fatty acids, the addition of cholesterol, the pH, the buffer selected, the divalent cations, and the lipoxygenase amount used [21,23,24]. Their advantages include their dynamic nature owing to the fact that they are composed of single-chain amphiphiles [21], and their retardation to thermal decomposition as compared to phosphatidylcholine liposomes [24]. Moreover, the presence of oleic acid enhances the permeability of these particles through different tissues [24], even superior in comparison to highly penetrable transfersomes. Owing to these advantages, ufasomes have been used in different studies as colloidal carriers for different antifungal agents [24,25], and for enhancing the topical penetration of drug molecules [23,26].

To the best of our knowledge, this is the first study evaluating the formulation of ITZ in unsaturated fatty acid-enriched vesicles and ufasomes. Accordingly, the aim of this study was to develop and evaluate ITZ-loaded ufasomes and evaluate their antifungal activity via in vitro microbiological tests. The prepared ITZ-loaded ufasomes were prepared using the thin layer hydration technique, and a full factorial design was employed to assess and optimize the different variables. Moreover, the produced ufasomes were morphologically examined using transmission electron microscopy (TEM), and the solid state was characterized using differential scanning calorimetry (DSC) and X-ray diffractometry (XRD). In addition, an in vitro examination of the microbiological effect of the optimized formula on *C. albicans* was conducted by estimating the minimum inhibitory concentration and using the agar well diffusion technique. Moreover, the effect of the optimized formula on the phospholipase A2, proteinase, interleukin 1 beta, and toll-like receptors-4 was examined on both the normal fibroblasts and the *C. albicans*-infected fibroblasts.

## 2. Materials and Methods

### 2.1. Materials

Itraconazole hydrochloride, cholesterol, sodium chloride, and sodium tetraborate were purchased from Sigma-Aldrich Chemical Co. (St. Louis, MO, USA). Oleic acid, methanol, and boric acid were purchased from Adwic, El-Nasr Pharmaceutical Co. (Cairo, Egypt). All other chemicals and solvents were of analytical grade and used as received.

### 2.2. Preparation of the ITZ-Loaded Ufasomes

ITZ-loaded ufasomes were prepared by the thin film hydration technique [27]. Accurately weighed amounts of oleic acid, ITZ, and cholesterol (if used) were placed in a rotatory flask of 1000 mL capacity, and then 50 mL of methanol was added to the flask and rotated till the formation of a homogenous solution using a rotary vacuum evaporator (Hoi-VAP, Heidolp, Germany). The flask was left to rotate at 150 rpm at 60 °C under vacuum until the methanol evaporation and film formation were complete. For the hydration of the film, 50 mL of borate buffer (pH 8.5) [28] was added to the rotary flask, and the rotatory evaporator was employed at 150 rpm and 60 °C without vacuum for one hour till the film’s total hydration. A sheer homogenizer (HG-15D, Daihan Scientific, South Korea) was further applied to the resulting suspension for 3 min at 100 rpm to ensure the homogeneity of the prepared ITZ-loaded ufasomes, as reported in Mittal et al. [23].

### 2.3. Evaluation of the Prepared ITZ-Loaded Ufasomes

#### 2.3.1. Determination of ITZ Percentage Encapsulation Efficiency (%EE)

The encapsulation efficiency percentages of ITZ in the prepared ITZ-loaded ufasomes were determined using the direct method. One mL of the prepared ITZ-loaded ufasomes was centrifuged using a cooling centrifuge (HermLe labortechnik GmbH, Berlin, Germany) for 1 min at 1000 rpm at room temperature. The residue was discarded to get rid of any un-entrapped ITZ, and then the sample was re-centrifugated at a rate of 15,000 rpm and a temperature of 4 °C for 120 min. The supernatant was discarded, and the precipitate was reconstituted in 50 mL methanol. Sonication was performed using an ultrasonic bath (UD50SH2LQ, RoHS, Bransonic, Shanghai, China) for 5 min till achieving a clear solution, which was then measured spectrophotometrically (Shimadzu, UV-1800, Japan) at the predetermined λmax based on the previously constructed standard curve. All measurements were done in triplicate at 25 °C, and the %EE was calculated as follows:

$$\%\mathrm{EE}=\frac{\mathrm{Measured}\;\mathrm{amount}\;\mathrm{of}\;\mathrm{ITZ}}{\mathrm{Theoretical}\;\mathrm{amount}\;\mathrm{of}\;\mathrm{ITZ}}\;\times \;100$$

#### 2.3.2. Determination of Particle Size and Polydispersity Index

Different formulations of ITZ-loaded ufasomes were examined using a zetasizer (Nano ZS-90 instrument, Malvern Panalytical Ltd., London, UK) to determine particle size (PS) and polydispersity index (PDI). The zetasizer utilizes the light scattering technique for size measurement [29].

About 0.1 mL of each preparation was diluted to 10 mL using distilled water and measured using the zetasizer, and each measurement was conducted 3 times at 25 °C.

#### 2.3.3. Zeta Potential

The zeta potential of ITZ-loaded ufasomes was measured to determine the overall charges acquired by the vesicles, which can be used to evaluate the stability of colloidal dispersions [30]. Samples of 0.1 mL of the formulations were diluted to 10 mL using distilled water and were measured using a zetasizer. Each measurement was performed in triplicate using a 90° scattering angle at 25 °C, dispersant viscosity of 0.89 cp, and a dielectric constant of 78.5. The viscosity of the samples was assumed to be equal to that of water [31].

### 2.4. Experimental Design

The ITZ-loaded ufasomes were prepared and optimized using a 2^2^3^1^ factorial design, followed by an analysis of variance (ANOVA) to determine the significance of each factor. The oleic acid amount (X_1_), the concentration of cholesterol (calculated as the percentage of cholesterol to oleic acid amount) (X_2_), and the drug amount (X_3_) were selected as the independent variables. The %EE (Y_1_), PS (Y_2_) and zeta potential (Y_3_) were selected as the dependent variables (responses) (Table 1). The base design consisted of 12 runs and the measured responses are shown in Table 2; the factor was considered significant at *p* ≤ 0.05.

### 2.5. Characterization of the Selected ITZ-Loaded Ufasomes

#### 2.5.1. Morphological Examination by Transmission Electron Microscope (TEM)

Morphological examination of the selected ITZ-loaded ufasomes was carried out using a transmission electron microscope (TEM, H-7500, Hitachi, Tokyo, Japan) [27]. This test is used to morphologically examine the size, sphericity, and aggregation of the ITZ-loaded ufasomes. Briefly, one drop of the vesicular dispersion was deposited on the surface of a carbon-coated copper grid, which was then allowed to dry at room temperature for 10 min for investigation by TEM [32].

#### 2.5.2. Differential Scanning Calorimetry (DSC)

The thermal properties of the pure ITZ, a physical mixture of the optimized formula (mixed at the ratio in the optimized formula), and the optimized lyophilized formula were assessed using DSC (DSC822e, Mettler-Toledo International Inc., Columbus, OH, USA). The optimized formula was converted to the lyophilized powder form prior to scanning, via freezing at −20 °C, followed by freeze-drying for 24 h in a freeze dryer (Novalyphe-NL 500; Savant Instruments Corp., Hicksville, NY, USA) until the complete sublimation of all solvents [27,33].

For DSC testing, 5 mg of previously lyophilized formula was accurately weighed and placed in sealed aluminum pans. The samples were heated at a scanning rate of 10 °C/min between 25 –400°C, with nitrogen as a blanket gas, and pure drug and physical mixture were used as a standard reference [25].

#### 2.5.3. X-ray Diffraction Study

The X-ray diffraction analysis (XRD) was conducted to study the crystalline state of the ITZ in the pure form and in the lyophilized optimized formula using an X-ray diffractometer (Bruker D8 advance diffractometer, Bruker Corporation, Billerica, MA, USA, Berlin, Germany) [34]. The XRD analysis of lyophilized selected ITZ-loaded ufasomes was conducted against the pure ITZ. The tested sample was applied over the sample holder using a thin spatula. Then, double-sided adhesive tape was applied over the sample holder. Then, the intensity of the diffracted beam was analyzed in the 2 θ range between 10° and 70° [35].

### 2.6. In Vitro Microbiology Evaluation on the Selected Formula on Candida albicans

#### 2.6.1. Strains Selection and Cultivation

*Candida albicans* ATCC 64,124 strain was the strain of choice for this study. Stock cultures of *C. albicans* strains were maintained in Sabouraud’s agar; and the cultures were propagated in liquid Sabouraud dextrose medium (SBD) at 30 °C for 12–16 h, till the strains reached the exponential growth phase. Activation of the culture was achieved in a 250 mL conical flask by inoculating a single colony from the SBD agar plate into 50 mL SBD broth. The flask was then incubated on an orbital shaking incubator (RADOBIO Scientific Co, Ltd-China) at 30 °C and 100 rpm for 24 h. Cells were harvested by centrifugation at 2000 rpm and washed thrice with 10 mM of sterile phosphate-buffered saline (PBS) with a pH of 7.4.

#### 2.6.2. Antifungal Activities Evaluation Using the Agar Well Diffusion Method

The antifungal activities of ITZ-loaded ufasomes F9 and pure ITZ were evaluated by the agar well diffusion method [36]. A volume of 100 μL of 0.5 MacFarland fungal suspension was spread on SBD. Wells were made on the agar plates using a sterile cork borer (Thermo Fischer Scientific, Saint Louis, MO, USA), and 100 μL of ITZ-loaded ufasomes and pure ITZ, both at a concentration of 1 mg/mL, were introduced separately to previously marked wells. The activity was evaluated by measuring the inhibition zone diameter, compared to 100 μL dimethyl sulfoxide (DMSO) as a negative control, after an incubation period of 48 h at 28 °C.

#### 2.6.3. Evaluation of the Minimum Inhibitory Concentration (MIC) of ITZ

The MIC of the ITZ-loaded ufasomes F9 was performed in a 96-well flat-bottomed microplate (Thermo Fischer Scientific, USA) by the micro-dilution method [37]. The initial inoculum was adjusted to 5 × 10^7^ CFU/mL, and the concentrations of the tested formulation were serially diluted in a range of 0.002–1 μg/mL.

Briefly, 50 μL of *C. albicans* was added to each well with 50 μL of serially diluted ITZ-loaded ufasomes F9 concentrations, to contain a total volume of 100 μL. An amount of 100 μL of *C. albicans* was used as a positive control, and 100 μL un-inoculated SBD broth media was used as a negative control. The microplate was incubated at 37 °C for 48 h, and absorbance was read at 620 nm using a microplate reader (Hidex Sense Microplate reader, Mustionkatu, Finland). The in vitro antifungal activity value was determined using the lowest drug concentration that showed 100% growth inhibition of the fungal strains. A negative sterility control plate was included in each run.

#### 2.6.4. Enzyme Secretion Assay

##### Proteinase Enzyme Secretion Assay

A proteinase enzyme secretion assay was conducted using the procedures stated by Santana et al. [38]. In brief, a casein solution was prepared by dissolving 10 mg of lyophilized casein in 5 mL of 50 mM borate buffer with a pH of 8.5, and then gently stirred till the full dissolution of casein protein using a magnetic stirrer at 100 rpm. Following that, 100 μL of the casein solution was added to a microplate well, and 100 μL of 50 mM of borate buffer was added to another microplate well as a blank.

For the evaluation, three groups were selected: 50 μL of the treatment group (MIC of ITZ-loaded ufasomes F9 with *Candida*-fibroblast cell culture), *Candida*-fibroblast cell culture (as positive control), and fibroblast cell culture (as negative control); and all three were added to both the casein and the blank wells separately. The plates were then incubated for 20 min at 37 °C, and 50 μL of 2,4,6-trinitrobenzene sulfonic acid (TNBSA) working solution was added to each well as a coloring agent. The plates were re-incubated for 20 min, and the absorbance of these wells was measured in a plate reader set to 450 nm (Beckman DU 64 UV-vis spectrophotometer, Beckman Coulter Lifesciences, USA). The change in absorbance at 450 nm (the absorbance generated by the proteolytic activity of protease) was calculated by subtracting the difference between the blank and the corresponding casein well. The standard curve used for the calculations was previously plotted at 450 nm against the trypsin concentration.

##### Phospholipase Enzyme Secretion Assay

For the phospholipase enzyme secretion assay, 50 μL of each group (treatment group (MIC of ITZ-loaded ufasomes F9 with *Candida*-fibroblast cell culture), *Candida* fibroblast cell culture (as positive control) and fibroblast cell culture (as negative control)) were added to each individual well, followed by the addition of 50 μL of substrate lipid mix to each well. A continuous reaction was then started by incubation at room temperature for 10 min and protection from light. Absorbance then was measured using a microplate reader at 515 nm. The standard curve used for the calculations was previously plotted at 515 nm.

#### 2.6.5. Expression of Interleukin 1β

Total RNA was isolated using a Qiagen tissue extraction kit according to the manufacturer’s instructions. Precisely 300 µL of the treatment group (MIC of ITZ-loaded ufasome F9 with *Candida*-fibroblast cell culture), *Candida*-fibroblast cell culture (as positive control), and fibroblast cell culture (as negative control) were added to lysis buffer RLT. Then the lysate was centrifuged for 3 min at 12,000 rpm, and the supernatant was carefully removed and transferred into a new micro-centrifuge tube. Following that, a volume of 350 µL of 70% ethanol was added to the cleared lysate. Then, 700 µL of the sample was transferred to an RNeasy spin column placed in a 2 mL collection tube and centrifuged for 15 s at ≥8000 rpm. In addition, a 700 µL Buffer RW1 was transferred to the RNeasy spin column and centrifuged for 15 s at ≥8000 rpm to wash the spin column membrane. Then, 500 µL of Buffer RPE was added to the RNeasy spin column and centrifuged for 15 s at ≥8000 rpm to wash the spin column membrane. Then, 500 µL of Buffer RPE was added to the RNeasy spin column and centrifuged for 2 min at ≥8000 rpm to wash the spin column membrane. Then the RNeasy spin column was placed in a new 1.5 mL collection tube. A volume of 30–50 µL RNase-free water was added directly to the spin column membrane and centrifuged for 1 min at ≥8000 rpm to elute the RNA. Then, the eluate was transferred to a new Eppendorf tube and stored at –80 °C for further use. The purity (A260/A280 ratio) and the concentration of RNA were obtained using a dual-wavelength Beckman spectrophotometer.

The total RNA (0.5–2 μg) was used for cDNA conversion using the high-capacity cDNA reverse transcription kit Fermentas. Volumes of 3 μL of random primers were added to the 10 μL of RNA, which was denatured for 5 min at 65 °C in the thermal cycler. The RNA primer mixture was cooled to 4 °C. Then, the cDNA master mix was prepared according to the kit instructions, as follows: 5 μL of first strand buffer, 2 μL of 10 mM dNTPs, 1 μL of RNase inhibitor (40 U/μL), 1 μL of MMLV-RT enzyme (50 U/μL), and 10 μL of DEPC-treated water. The total volume of the master mix was 19 μL for each sample. This was added to the 13 μL RNA-primer mixture resulting in 32 μL of cDNA. The last mixture was incubated in the programmed thermal cycler for one hour at 37 °C followed by the inactivation of enzymes at 95°C for 10 min, and finally cooled at 4 °C. Then, the RNA was changed into cDNA. The converted cDNA was stored at –20 °C.

Real-time PCR amplification and analysis were performed using the real-time PCR Step One apparatus (Applied Biosystem software version 7.1, USA), where the assay with primer sets was optimized at the annealing temperature; the primer sequence for expression of interleukin one β was (F) ACAGATGAAGTGCTCCTTCCAG and reverse (R) CATGGCCACAACAACTGACG were used for the amplification of IL-1β and the housekeeping gene, ACTB (F 5′-AGCGAGCATCCCCCAAAGTT-3′ and R: 5′-GGGCACGAAGGCTCATCATT-3′).

The SYBR GREEN 1 reaction master mix for Q-PCR was prepared in which each sample was prepared as 1 μL of the forward primer, 1 μL of the reverse primer, 12.5 μL of Syber green mix, 5 μL of cDNA template, and 5.5μL of RNAse free water, for a total volume of 25 µL.

The relative quantization was calculated according to Applied Bio^®^ system software version 7.1 using the following equations:

∆Ct = Ct gene test − Ct endogenous control (housekeeping)

∆∆Ct = ∆Ct sample − ∆Ct control

RQ = Relative quantification = 2 − ∆∆Ct

where RQ is the fold change compared to the calibrator (untreated sample).

#### 2.6.6. Flow Cytometry Analysis of TLR-4

The antigen activation of toll-like receptors TLR-4 was achieved in vitro by stimulating fibroblast cells to produce cytokines by *Candida*-fibroblast cell culture [39]. Then the stimulated cells were fixed by using BD Cytofix/Cytoperm™ solution, followed by the permeabilization of the fixed cells by washing them two times in 1× BD Perm/Wash buffer (e.g., 1 mL/wash for staining in tubes and 250 µL/wash final volume for staining in microwell plates). A concentration of 10× BD Perm/Wash buffer was diluted in distilled H_2_O to make a 1× solution prior to use. The staining of intracellular cytokines was performed by wash cells two times with 1× BD Perm/Wash buffer (1 mL/wash for staining in tubes) and 250 µL/wash final volume for staining in microwell plates of the three groups—MIC of ITZ-loaded ufasomes F9 with *Candida*-fibroblast cell culture, *Candida* fibroblast cell culture (as positive control) and fibroblast cell culture (as negative control)—and resuspended in a staining buffer prior to flow cytometric analysis using a FACS Callibur™ (BD Immunocytometry Systems, USA) and CellQuest software (BD Biosciences-USA).

## 3. Results and Discussion

### 3.1. Preparation of ITZ-Loaded Ufasomes

ITZ-loaded ufasomes were prepared by the thin film hydration method, in which the organic solvent used is quickly evaporated to form a film. The film is then immediately hydrated by a buffer with a pH of 8.5, which is the most suitable condition to form the ufasome [40]. The biocompatibility stems back to the presence of oleic acid, due to its ability to form the vesicular structures in an aqueous solution [41]. Several researchers have used this method, including Bhattacharya S. [24], who prepared terbinafine-loaded ufasomes using the thin film hydration technique to get the highest drug entrapment efficiency (52.45 ± 0.56%), in addition to the desired globular size [24,42]. The thin film hydration technique is preferred as a result of its simplicity, practicability, and its ability to give mostly uniform particles [43].

### 3.2. Evaluation of ITZ-Loaded Ufasomes

#### 3.2.1. Effect on the Tested Formulation Variables on %EE

Table 2 represents the average %EE of ITZ in the prepared ITZ-loaded ufasomes. The results revealed high entrapment of ITZ in the prepared ufasomes, with %EE over 80% in all the prepared ufasomes. This could be attributed to the high affinity of the highly lipophilic drug (ITZ) to the lipophilic carrier (oleic acid), which is supported by numerous studies [44,45,46].

**Figure 1:**
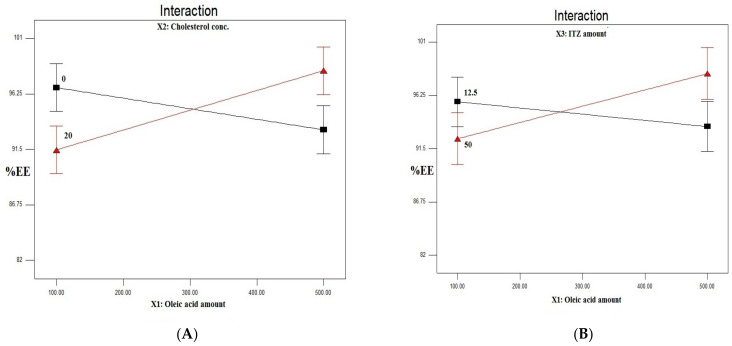

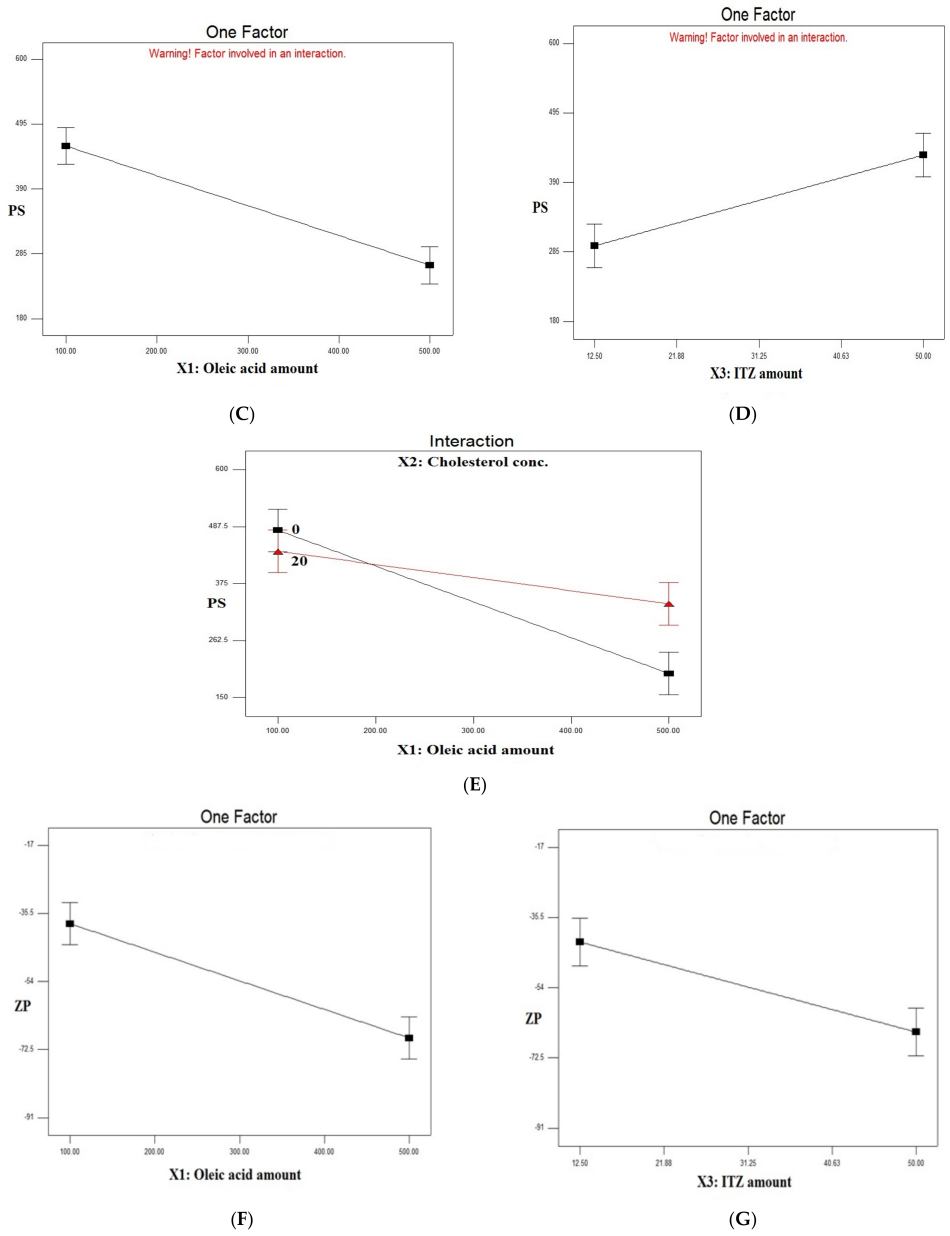
Line chart showing the effect of: (**A**) oleic acid amount–cholesterol concentration interaction (**A**,**B**) on %EE of ITZ, (**B**) oleic acid amount–drug amount interaction (**A**–**C**) on %EE of ITZ, (**C**) oleic acid amount (X1) on the PS of ITZ-loaded ufasomes, (**D**) drug amount (X3) on the PS of the ITZ-loaded ufasomes, (**E**) oleic acid amount–cholesterol concentration interaction (**A**,**B**) on the PS of ITZ-loaded ufasomes, (**F**) oleic acid amount (X1) on the ZP of ITZ-loaded ufasomes, and (**G**) drug amount (X3) on the ZP of ITZ-loaded ufasomes. Abbreviations: ITZ: itraconazole, %EE: percentage encapsulation efficiency, PS: particle size, ZP: zeta potential.

There was an observed significant difference (*p* = 0.0009) in the interaction of oleic acid and cholesterol concentration. Figure 1A shows the interaction effect of the oleic acid amount and the cholesterol concentration on the %EE of ITZ in the prepared ITZ-loaded ufasomes. In the absence of cholesterol, there was a significant decrease in the %EE with the increase of the oleic acid amount. This could be attributed to the decrease in the molar ratio of the ITZ to the oleic acid, or the increase in the oleic acid to ITZ ratio. These results were shared with several scientists [25,47]. In their study on oleic acid vesicles for the topical delivery of fluconazole, Zakir et al. [48] stated that the mean entrapment efficiency of their developed vesicles increased with the increase in the molar quantity of fluconazole up to a 7:3 oleic acid to fluconazole ratio.

On the other hand, at a cholesterol level of 20%, a significant increase in the %EE upon the increase in the oleic acid amount was observed (Figure 1A). Cholesterol has the function of modulating the fluidity, elasticity, and permeability of the vesicle. This results in rather large imperfections in the lattice, leading to the presence of enough space for the drug molecules to be encapsulated [49]. This finding is in accordance with Emami et al. [50], who observed that increasing the percentage of oleic acid in their nanostructured lipid carriers (NLCs) containing cholesterol led to an increase in the %EE of the drug (paclitaxel). Moreover, Uprit et al. [51] observed that increasing the amount of oleic acid in their NLCs from 50 to 75 and 100 mg, led to a significant increase in the %EE of minoxidil in the presence of the same amount of cholesterol (0.015 mg).

A significant interaction (*p* = 0.0252) between the oleic acid amount and the drug amount was also observed (Figure 1B). At low oleic acid amounts, increasing the drug amount from 12.5 to 50 mg resulted in a lower %EE of ITZ in the prepared ITZ-loaded ufasomes. This could be attributed to the lower amount of ufasomes developed at a low oleic acid amount (100 mg), which was not available to accommodate the higher drug amount. On the other hand, increasing the drug amount at a high oleic acid amount (500 mg) resulted in a higher %EE of ITZ owing to the presence of more ufasomes developed at a high oleic acid amount, which were sufficient to entrap the higher drug amount.

#### 3.2.2. Effect of the Tested Formulation Variables on PS and PDI

The measured average PS values of the different ITZ-loaded ufasomes are presented in Table 2, where the results ranged from 185 ± 3 to 570 ± 19 nm. Figure 1C shows the effect of oleic acid amount on the PS of the prepared ITZ-loaded ufasomes. Increasing the oleic acid amount led to a significant decrease (*p* < 0.0001) in the PS of the prepared ITZ-loaded ufasomes. This is in accordance with the results obtained by Mittal et al. [27,52], who reported that an increase of the oleic acid amount will result in the reduction of vesicle size owing to the formation of micelles, not vesicles.

Figure 1D shows the effect of drug amount on the PS of the prepared ITZ-loaded ufasomes. Increasing the ITZ amount significantly (*p* = 0.0004) increased the PS of the developed ufasomes, owing to the increase of the drug loading inside the prepared ufasomes. This is in good agreement with the results obtained by Salama and Aburahma [27], who observed that there was a proportional relationship between the cinnarizine concentration and the PS of the developed ufasomes containing the cinnarizine.

A significant interaction (*p* = 0.0038) between the oleic acid amount and the cholesterol concentration was observed. Figure 1E shows the interaction effect of oleic acid amount and the cholesterol concentration on the PS of the prepared ITZ-loaded ufasomes. In absence of cholesterol (0%), increasing the oleic acid amount decreased the PS of the prepared ufasomes to a greater extent compared to that in the presence of cholesterol.

The PDI results of ITZ-loaded ufasomes are presented in Table 2. The values of PDI represent the size distribution inside the formulation, and range between 0 and 1. However, it is desirable for them to be lower than 0.5 in order to ensure a monodisperse population and homogeneity [53]. The PDI values of the prepared ITZ-loaded ufasomes ranged from 0.179 to 0.834. It was observed that most of the samples had PDI values lower than 0.5 (except formulae F2, F4, and F5), indicating that the method of preparation was appropriate and reproducible [54,55].

#### 3.2.3. Effect of the Tested Formulation Variables on ZP

Zeta potential is considered an important factor for predicting the stability of the formulation [56], as it represents the electrostatic repulsion between the particles in the dispersion [57]. For instance, when the attractive forces are greater than the repulsion forces, the particles will attract and flocculate. This means the end of the stability of the dispersion, and that is the case with small ZP values. However, if the ZP values are high (either positive or negative), this indicates that the formulation is stable [58]. The drug delivery system is considered stable in case the ZP value is more than −30 mV as the electrical repulsion among the system particles prevents their aggregation [59]. Table 2 illustrates the numerical ZP values. It was noticed that all the prepared ITZ-loaded ufasomes carried a negative charge, with most of them having a high negative charge (>−30 mV), indicating the excellent stability of these ufasomes [59].

Figure 1F shows the effect of oleic acid amount on the ZP of the developed ITZ-loaded ufasomes. Increasing the oleic acid amount led to a significant increase (*p* < 0.0001) in the ZP (it became more negative). This is in accordance with the results obtained by Levy et al. [60], who observed that an increase in the oleic acid amount used in their prepared diazepam submicron emulsion caused an increase in the ZP. Moreover, Washington and Davis [61] reported that increasing the oleic acid to the emulsion resulted in higher resistance to charge potential, and thus a higher ZP.

Figure 1G shows the effect of drug amount on the ZP of the developed ITZ-loaded ufasomes. Increasing the drug amount significantly decreased (*p* = 0.0013) the ZP value (a more negative value). This is in accordance with the results obtained by Feng et al. [62], who found that the increase in the drug inside the chitosan-ricinoleic acid loaded with rotenone resulted in increasing the ZP. They linked the increase in the amount of negative charge to the increase in the amount of the drug.

Based on the obtained results, and the conditions for the selection of the best-achieved formulation using Design Expert^®^ software (version 7), the best-achieved formula was ITZ-loaded ufasomes F9, with the highest desirability value of 0.850, and thus was used for further investigation.

### 3.3. Evaluation of the Best Achieved ITZ-Loaded Ufasomes

#### 3.3.1. Morphological Examination Using TEM

**Figure 2:**
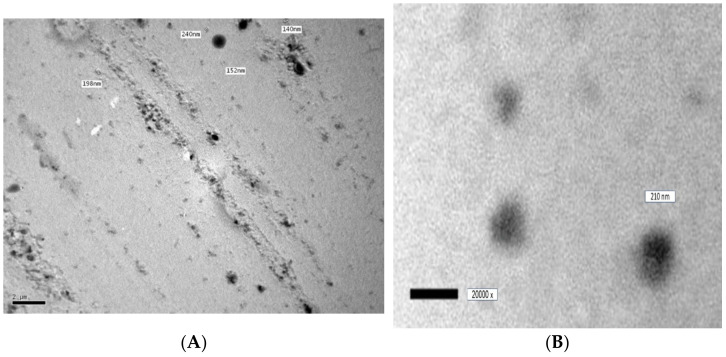
TEM photomicrograph of the optimized ITZ-loaded ufasomes (F9), at (**A**) 3000×, and (**B**) 20,000×.

The photomicrograph produced using TEM for the best-achieved formula F9 is presented in Figure 2. It was observed that the ufasomes were unilamellar with uniform spherical discreet shapes with neither fusion nor aggregation. In addition, the diameter of the vesicles observed by the TEM was in accordance with the data obtained by the PS analyzer.

#### 3.3.2. Differential Scanning Calorimetry (DSC)

**Figure 3:**
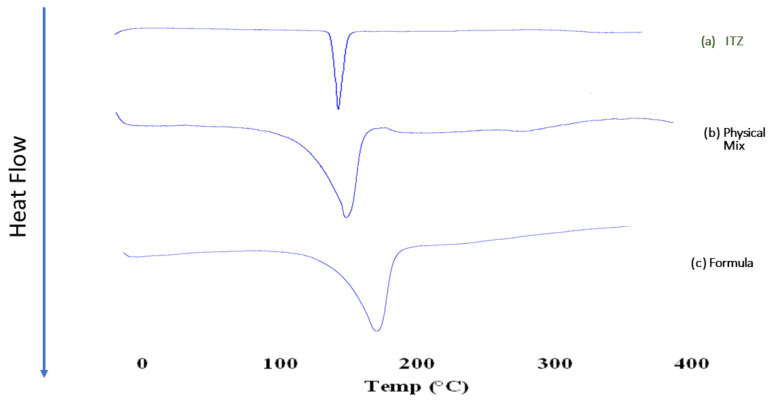
DSC thermogram of (**a**) pure ITZ, (**b**) physical mixture of the drug and oleic acid (1:10 *w*/*w*), (**c**) lyophilized ITZ-loaded ufasomes F9. Abbreviations: ITZ: itraconazole.

DSC is a tool used to determine the possible interactions between the drug and the excipients [23]. Figure 3 shows the DSC thermogram of the pure form of the drug, the physical mixture of the drug and oleic acid (1:10 *w*/*w*), and finally, the optimized lyophilized formula. ITZ exhibited an endothermic peak at 166.2 °C, corresponding to its melting point [63]. The DSC of the physical mixture did not change, due to the lack of interaction between the ITZ and the oleic acid. In the case of the lyophilized optimized formula (F9), the significant peak of ITZ was retained. The reason behind that is the absence of interaction and there was no formation of inclusion complexes, which was in accordance with the results obtained by Shehatta et al. [63].

#### 3.3.3. X-ray Diffraction Study

**Figure 4:**
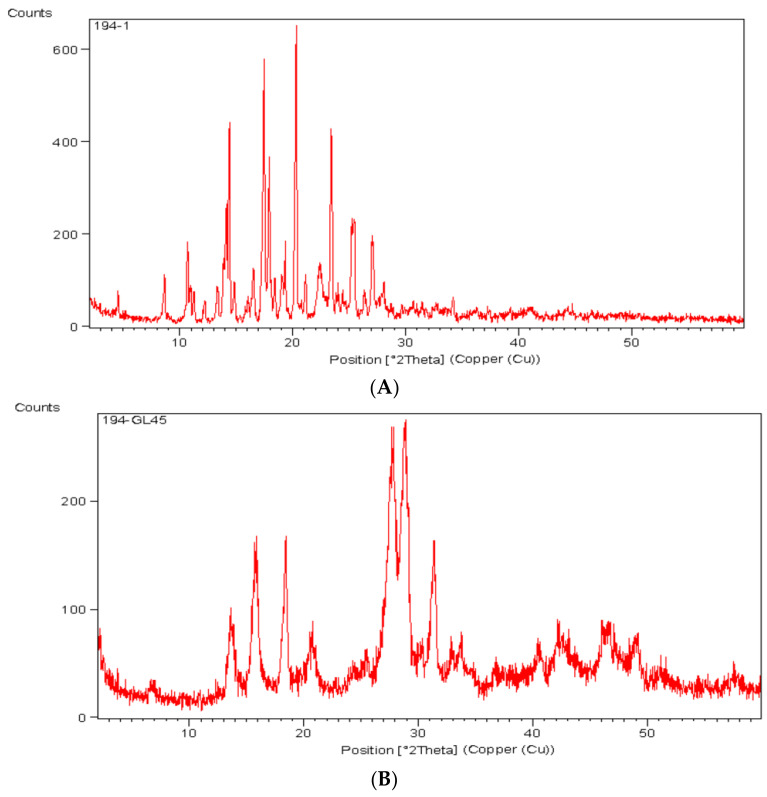
X-ray diffractogram of (**A**) pure ITZ, (**B**) lyophilized ITZ-loaded ufasomes F9. Abbreviations: ITZ: itraconazole.

The XRD study was performed for pure ITZ and the optimized lyophilized ITZ-loaded ufasomes (F9), and the diffractograms are shown in Figure 4. ITZ is crystalline in nature with sharp peaks at 2 θ 10.94°, 14.13°, 16.20°, 17.48°, 18.38°, 21.12°, 25.32°, and 28.05°, corresponding to ITZ significant peaks as represented in Figure 4A [64]. These peak intensities were reduced and shifted in the diffractogram of the lyophilized ITZ-loaded ufasomes (Figure 4B). This could be attributed to the encapsulation of the ITZ inside the vesicle, which changed ITZ’s nature. In addition, the reduction in the number of peaks in the diffractogram of the ufasomes explains that the ITZ lost part of its physical nature when it was successfully incorporated into the ufasomes [65].

Upon these and the previous findings, F9 was selected for further in vitro antifungal activity and microbiological assessments.

### 3.4. In Vitro Microbiology Evaluation of the Selected Formula on Candida albicans

#### 3.4.1. Strain Selection and Cultivation

The *C. albicans* ATCC 64,124 strain was used in this study due to the activity of ITZ on this strain of *Candida* [66]. *C. albicans* is the fungus generally considered responsible for most topical infections. ITZ had a great impact against *Candida* in comparison to other antifungals [67]. Therefore, *Candida* was used for evaluating the efficacy of the ITZ. Sabouraud dextrose growth medium (SBD) was used to cultivate the fungal infection, as it is one of the most common media of choice in the literature [68,69,70].

#### 3.4.2. Antifungal Activity Evaluation Using the Agar Well Diffusion Method

**Figure 5:**
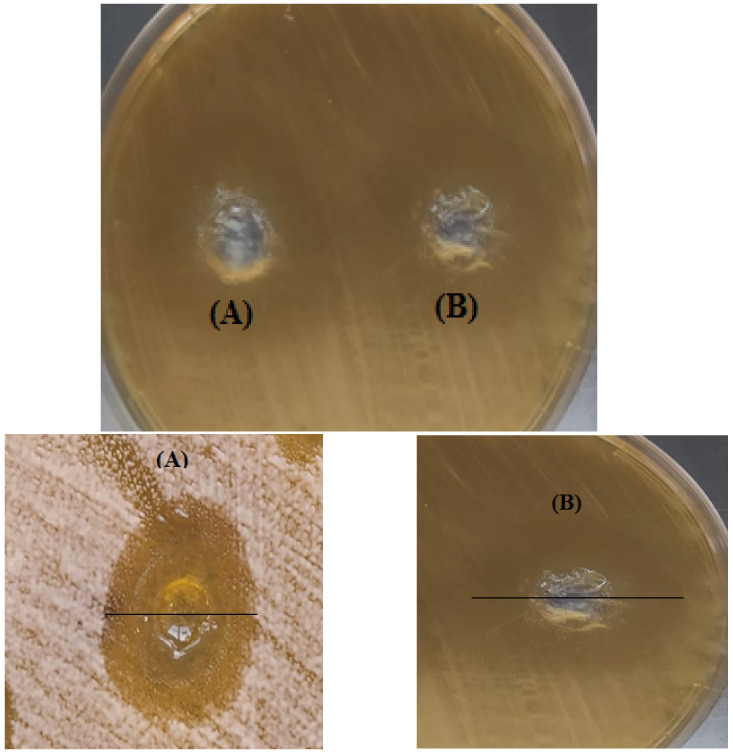
Agar well diffusion photograph of (**A**) ITZ-loaded ufasomes F9, (**B**) pure ITZ, against *C. albicans,* together and individually. Abbreviations: ITZ: itraconazole.

A susceptibility assay of ITZ-loaded ufasomes F9 showed antifungal activity against *C. albicans* using the agar well diffusion assay (Figure 5). The agar well diffusion method was developed for the first time in 1940 [71]. It is used for antimicrobial susceptibility testing as an in vitro method [72]. In this method, the ITZ diffuses from the holes into the agar where it shows anti-fungal efficacy [73]. This method was used to compare the efficacy of our formula against the pure ITZ. It was observed that the ITZ-loaded ufasomes F9 diameter was 25 mm, while that of the pure ITZ was only 15 mm. This difference could be attributed to the presence of oleic acid, which exhibits antifungal activity. Walters et al. [74] studied the effect of oleic acid against plant pathogenic fungi, and they observed that oleic acid had antifungal activity against both Pythium ultimum and Pyrenophora avenae. In addition, Muthamil et al. [75], in their recent proteomic analysis of oleic acid against *C. albicans* virulence and biofilm formation, have observed that oleic acid-induced oxidative stress responses, targeting proteins involved in glucose metabolism, lipase production, and amino acid biosynthesis in the fungi, emphasizing the anti-virulence effect of oleic acid.

#### 3.4.3. Evaluation of the Minimum Inhibitory Concentration (MIC) of ITZ

The minimum inhibitory concentration (MIC) value is the lowest concentration of an anti-fungal which in controlled in vitro conditions completely prevents the visible growth of the tested strain [76]. In this test, ITZ-loaded ufasomes F9 had caused visual inhibition of *C. albicans* at levels of 0.0625–0.125 μg/mL. The results showed good fungicidal activity when compared with ITZ as control. These results are in accordance with several studies conducted on the MIC of ITZ. Bueno et al. [77] observed that ITZ alone had a range of MIC 0.125–1 μg/mL against different species of *Candida*. Moreover, Corderiro et al. [78] tested the MIC of ITZ against several species of *C. albicans*, and found that the MIC ranges from 0.031125 to 16 mg/L. In addition, Erum et al. [79] observed that the MIC of ITZ on *C. albicans* was 0.0625–0.125 μg/mL.

#### 3.4.4. Enzyme Secretion Assay

The rationale for studying the activity of proteinases and phospholipases was that such hydrolytic enzymes have been reported to be secreted by *C. albicans* and are known to elicit host tissue damage [79,80,81]. *C. albicans* can synthesize as partyl proteinase through the destruction of elastin, which is involved in the invasion of host tissues. Additionally, phospholipases are responsible for the hydrolysis of phospholipids, which help *Candida* to invade the cells and destroy the host cell membrane [81,82]. This study sought to identify any modulatory effects of ITZ-loaded ufasomes F9 on the enzyme activity on *C. albicans*, specifically that of extracellular enzymes, which play a pivotal role in the binding, invasion, and destruction of the host cellular structure [83].

##### Proteinases Enzyme Secretion Assay

**Figure 6:**
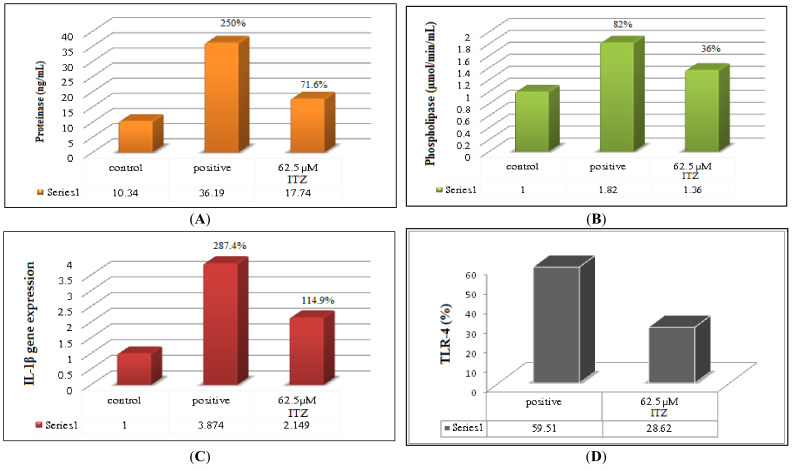
Bar charts illustrating the effect of the MIC of ITZ-loaded ufasomes F9 with *C. albicans*-fibroblast cell culture, *C. albicans*-fibroblast cell culture (positive) and fibroblast cell culture (negative) effects on (**A**) proteinase enzyme secretion, (**B**) phospholipase enzyme secretion, (**C**) IL-1β pro-inflammatory cytokines gene expression using real-time PCR, and (**D**) TLR-4 flow cytometry analysis. Abbreviations: MIC: minimum inhibitory concentration, ITZ: itraconazole, IL: interleukin, PCR: polymerase chain reaction, TLR-4: toll-like receptors-4.

The results of the proteinase enzyme secretion assay are represented as a bar chart in Figure 6A. It was observed that there was a reduction in the proteinase enzyme for the ITZ-loaded ufasomes (F9) formula against the positive control, with the levels decreasing from 36.19 ng/mL (250%) to 17.74 ng/mL (71.6%) for the group treated with ITZ. This inhibition of the virulence factor proteinase enzyme is responsible for showing activity against the biofilm formation of *Candida*, suggesting that ITZ-loaded ufasomes F9 formula is both fungicidal and anti-biofilm formation against *C. albicans* [84,85].

##### Phospholipase Enzyme Secretion Assay

The results of the phospholipase enzyme secretion assay are represented as a bar chart in Figure 6B. It was observed that the group treated with ITZ-loaded ufasomes F9 showed an observed reduction (1.36 μmol/min/mL with a % error of 36%) in the phospholipase secretion in comparison to the positive control group (1.82 μmol/min/mL with % error of 82%), and closer in result to the negative control (1.0 μmol/min/mL). Such findings suggest that the ufasomes formula decreased the phospholipase enzyme by inhibiting its production. These findings were in accordance with Zuza-Alves et al. [86] in their study of *Candida tropicalis*. They suggested that the antifungal activity inhibition is attributed to proteinase and phospholipase production inhibition. These findings could suggest that ITZ is significantly potent in inhibiting fungal enzymes proteinase and phospholipase [85].

#### 3.4.5. Expression of Interleukin 1β

The co-culture model was used in this study to provide valuable information on IL-1β pro-inflammatory cytokines gene expression in our three selected groups using real-time PCR-quantified host gene expression of IL-1β pro-inflammatory cytokines, and the results are illustrated in Figure 6C. It was observed that the group treated with *C. albicans* showed a significant increase (3.874-fold with a % error of 287.4%) in IL-1β expression, in comparison to the group treated with fibroblast cells (1-fold). These results are due to the IL-1β activation upon the presence of *C. albicans*. Similar results were observed by Zhang et al. [87] in their study of the effects of different fungi on IL-1β expression in mouse dendritic cells. They observed that *C. albicans* had a significant inflammation-inducing effect in a concentration and time-dependent pattern.

On the other hand, in the *C. albicans*-fibroblast-infected group treated with 0.0625 μg/mL ITZ-loaded ufasomes F9, there was an observed significant decrease (2.149-fold with % error of 114.9%) in IL-1β expression. This is attributed to the presence of ITZ, which enhanced the effect of IL-1β on the *C. albicans* infection, leading to the lower secretion of cytokines. Similar results were observed by Kullberg et al. [88] in their study of the combined effect of fluconazole and recombinant interleukin-1 on systemic candidiasis in neutropenic mice. They stated that the combined treatment with both IL-1 and fluconazole caused a significant decrease in the numbers of *C. albicans*, in comparison to IL-1 treatment alone. Moreover, Shin et al. [89] observed that ITZ treatment in the lipopolysaccharide-induced acute lung injury in rats caused a reduction in the elevated BAL fluid levels of IL-1β levels, in comparison to the control saline group. Moreover, the presence of oleic acid might have reduced the IL-1β expression. Similar results were observed by Yu et al. [90] in their study of the effect of oleic acid-based nanosystems on respiratory distress syndrome. They stated that the oleic acid-based nanoparticles with the large size and greater amount of oleic acid achieved a greater reduction in the IL-6 levels than those smaller ones.

#### 3.4.6. Flow Cytometry Analysis of TLR-4

The results of the TLR-4 flow cytometry analysis are represented as bar chart in Figure 6D. TLRs are transmembrane proteins on the surface of immune cells, which detect conserved molecular motifs known as “microbe-associated molecular patterns” from various organisms. They interact with several adapter proteins to activate transcription factors, leading to the production of inflammatory cytokines and the activation of adaptive immunity [91]. The results illustrate the downregulation of TLR-4 in the ITZ-loaded ufasomes F9 fibroblast group infected with *C. albicans* (28.62%), in comparison with fibroblast infected with *C. albicans* (59.51%). While the exact roles of TLRs for different fungal species are not clear yet, it was observed that TLR-4 was upregulated during the fungal infection [92,93]. TLRs have been implicated in mediating host immune responses during systemic candidiasis [94,95]. However, upon the addition of an anti-bacterial or anti-fungal agent, the TLR-4 expression is downregulated, due to the effect of this agent on the reduction in the virulence of the fungi. Similar results were observed by Semlali et al. [96]. They evaluated the effect of their synthetic formulated anti-microbial KSL-W and amphotercin-B on different immunomodulatory factors. They observed that both agents had the ability to significantly downregulate the TLR-4 mRNA expression in the *C. albicans*-infected epithelial cells, compared to the *C. albicans*-infected cells with no treatment, in a dose-dependent manner. They attributed this to the ability of each of those agents to reduce *C. albicans* virulence, leading to the reduction in TLR-4 expression.

## 4. Conclusions

In the present study, ITZ-loaded ufasomes were successfully prepared using the thin film hydration technique, employing a 2^2^3^1^ full factorial design. The optimized formula using Design Expert software had a high %EE, high negative ZP value, and a small PS, with spherical unilamellar morphology. DSC and XRD were used to ensure the absence of any interactions between ITZ and the used materials. Moreover, the microbiology study confirmed the ability of the produced ITZ-loaded ufasomes to inhibit the phospholipase and proteinase secretion by the *C. albicans*, and the downregulation effect on the immunomodulatory IL-1β and TLR-4, making the proposed formula a promising system for the treatment of *C. albicans* infections.

## Figures and Tables

**Table 1:** Design parameters and constraints for the factorial design of ITZ-loaded ufasomes.

Independent Variables	Levels of Variables
X1: Oleic acid amount (mg)	100.0 500.0
X2: Cholesterol concentration (%)	0.00 20.0
X3: Drug amount (mg)	12.5 25.0 50.0
**Responses**	**Constraints**
Y1: %EE	Maximum
Y2: PS	Minimum
Y3: ZP	Maximum

Abbreviations: ITZ: itraconazole, %EE: percentage encapsulation efficiency, ZP: zeta potential.

**Table 2:** Formulations of the experimental design and their response results.

Formula Number	Independent Variables			Dependent Responses			
	X1: Oleic Acid Amount (mg)	X2: Cholesterol Concentration (%)	X3: Drug Amount (mg)	Y1: %EE * (%)	Y2: PS * (nm)	Y3: ZP * (mV)	PDI *
F1	100	0	12.5	98.9 ± 0.2	280 ± 5	−18 ± 0.8	0.44 ± 0.0
F2	100	0	25	96.1 ± 0.5	558 ± 29	−49 ± 0.3	0.64 ± 0.1
F3	100	0	50	95.7 ± 0.9	570 ± 19	−52 ± 0.4	0.53 ± 0.1
F4	100	20	12.5	96.2 ± 0.2	268 ± 10	−31 ± 0.7	0.70 ± 0.0
F5	100	20	25	87.7 ± 0.4	544 ± 39	−19 ± 0.3	0.83 ± 0.1
F6	100	20	50	90.9 ± 1.3	467 ± 13	−51 ± 0.3	0.37 ± 0.0
F7	500	0	12.5	95.3 ± 0.6	208 ± 3	−78 ± 0.4	0.18 ± 0.0
F8	500	0	25	83.6 ± 0.6	185 ± 3	−22 ± 0.3	0.27 ± 0.0
F9	500	0	50	99.4 ± 0.7	190 ± 1	−82 ± 0.4	0.29 ± 0.0
F10	500	20	12.5	95.6 ± 0.9	314 ± 2	−66 ± 0.4	0.42 ± 0.0
F11	500	20	25	100.5 ± 0.1	230 ± 5	−71 ± 0.2	0.41 ± 0.1
F12	500	20	50	98.3 ± 0.1	445 ± 8	−90 ± 0.2	0.47 ± 0.0

* Data are presented as mean (*n* = 3), ± standard deviation. Abbreviations: %EE: percentage encapsulation efficiency, ZP: zeta potential, PDI: polydispersity index.

## Data Availability

Data are available upon request.
